# Perceptions Underlying Addictive Technology Use Patterns: Insights for Cognitive-Behavioural Therapy

**DOI:** 10.3390/ijerph19010544

**Published:** 2022-01-04

**Authors:** Olatz Lopez-Fernandez, Lucia Romo, Laurence Kern, Amélie Rousseau, Pierluigi Graziani, Lucien Rochat, Sophia Achab, Daniele Zullino, Nils Inge Landrø, Juan José Zacarés, Emilia Serra, Mariano Chóliz, Halley M. Pontes, Mark D. Griffiths, Daria J. Kuss

**Affiliations:** 1 Fundación Jiménez Díaz University Hospital Health Research Institute, Fundación Jiménez Díaz University Hospital, Avda. Reyes Católicos 2, 28040 Madrid, Spain; 2 CLInique PSYchanalyse Développement (CLIPSYD—EA4430), Université Paris Nanterre, 200 Av. de la République, 92000 Nanterre, France; lromodes@parisnanterre.fr; 3 Hôpital Universitaire Raymond Poincaré, AP-HP Garches, CESP, U1018 INSERM UPS UVSQ 2, 104 Bd Raymond Poincaré, 92380 Garches, France; 4 EA 2931, Centre de Recherches sur le Sport et le Mouvement (CESRM), Université Paris Nanterre, 200 Av. de la République, 92000 Nanterre, France; laurence.kern@gmail.com; 5 Psychology Department, Centre d’Etudes et Recherches en Psychopathologie et Psychologie de la Santé (EA7411), Université Toulouse Jean Jaurès, 5 All. Antonio Machado, 31058 Toulouse, France; amelie.rousseau@univ-tlse2.fr; 6 Laboratoie VCR, Ecole de Psychologues Praticiens de l’Institut Catholique de Paris, 71, Rue Molière, 69000 Lyon, France; 7 LPS EA 849, Aix-Marseille University, Jardin du Pharo, 58 Boulevard Charles Livon, 13007 Marseille, France; pierluigi.graziani@free.fr; 8 Psychologie, Langues, Lettres et Histoire Département, University of Nîmes, Rue de Docteur Georges Salang Cs 13019, 30021 Nîmes, France; 9 Specialized Facility in Behavioral Addiction ReConnecte, Department of Mental Health and Psychiatry, University Hospitals of Geneva, Rue Gabrielle-Perret-Gentil 4, 1205 Geneva, Switzerland; Lucien.Rochat@unige.ch; 10 Psychological and Sociological Research and Training Unit, Department of Psychiatry, University of Geneva, 24 Rue du Général-Dufour, 1211 Geneva, Switzerland; sophia.achab@hcuge.ch (S.A.); daniele.zullino@hcuge.ch (D.Z.); 11 Outpatient Treatment Unit for Addictive Behaviors ReConnecte, Geneva University Hospitals, Rue Gabrielle-Perret-Gentil 4, 1205 Geneva, Switzerland; 12 Clinical Neuroscience Research Group, Department of Psychology, University of Oslo, Forskningsveien 3A, 0373 Oslo, Norway; n.i.landro@psykologi.uio.no; 13 Department of Developmental and Educational Psychology, University of Valencia, Av. de Blasco Ibáñez, 13, 46010 Valencia, Spain; juan.j.zacares@uv.es (J.J.Z.); emilia.serra23@gmail.com (E.S.); 14 Department of Basic Psychology, University of Valencia, Av. de Blasco Ibáñez, 13, 46010 Valencia, Spain; Mariano.Choliz@uv.es; 15 Department of Organizational Psychology, Birkbeck, University of London, Malet St, London WC1E 7HX, UK; contactme@halleypontes.com; 16 International Gaming Research Unit, Cyberpsychology Research Group, Psychology Department, Nottingham Trent University, 50 Shakespeare Street, Nottingham NG1 4FQ, UK; mark.griffiths@ntu.ac.uk (M.D.G.); daria.kuss@ntu.ac.uk (D.J.K.)

**Keywords:** cognitive-behavioural therapy, cognition, behaviours, internet addiction, compulsive internet use, internet use-related addiction, adults, preoccupied attachment style, mixed-methods

## Abstract

Cognitive-Behavioural Therapy (CBT) is considered the ‘gold standard’ in the treatment of addictive disorders related to excessive technology use. However, the cognitive components of problematic internet use are not yet well-known. The aim of the present study was to explore the cognitive components, that according to problematic users, can lead to potential internet addiction. A total of 854 European adults completed an online survey using a mixed-methods design. Internet problems and attachment styles were assessed, prevalence rates estimated, correlations, chi-squared automatic interaction detection, and content analysis were performed. Self-reported addictions to social networking, internet, and gaming had a prevalence between 1.2% (gaming) to 2.7% (social networking). Self-perception of the addiction problem and preoccupied attachment style were discriminative factors for internet addiction. In an analysis of qualitative responses from self-identified compulsive internet users, a sense of not belonging and feeling of disconnection during life events were perceived as causes for internet addiction. The development depended on a cycle of mixed feelings associated with negative thoughts, compensated by a positive online identity. The severity of this behaviour pattern produced significant impairment in various areas of the participants’ functioning, suggesting a possible addiction problem. It is suggested that health professionals administering CBT should target unhealthy preoccupations and monitor mixed feelings and thoughts related to internet use to support coping with cognitive distortions.

## 1. Introduction

Cognitive-Behavioural Therapy (CBT) has been widely adopted as a type of psychological treatment to various mental health disorders, including addiction problems, by focusing on users’ cognitions (e.g., thoughts, beliefs, mental schemas, and metacognitions) as the main driver of effective emotional and behavioural regulation using different strategies [1]. These interventions emerged during the second half of the 20th century and evolved in the 1990s alongside Mindfulness-Based Therapies (i.e., Mindfulness-Based Relapse Prevention [MBRP], Acceptance and Commitment Therapy [ACT], Dialectical Behavioural Therapy [DBT]). These treatment approaches seek to help users build self-confidence and address problematic cognitions which are at the root of the addiction problems (e.g., recognising their triggers). However, there is still a gap in knowledge regarding what these underlying cognitions are in emergent addiction disorders, especially those related to the use of current technologies.

In the CBT approach, the key underlying assumption is that users’ reactions to the addiction object are learned, and the path to recovering wellbeing relates to ‘unlearning’ the addictive behaviours, suggesting that the developmental pathway can be reversed. The first step consists of managing present beliefs linked to thoughts (e.g., negative views of self, others, or the future). Subsequently, the addictive behaviours and feelings linked to both, internal and external factors that may have had a role in generating or maintaining the addictive usage patterns and identifying the set of vulnerabilities the individual presents with should be targeted. This procedure can lead to progressively re-establishing healthy behaviours. One of the most relevant aspects to increase understanding is to discern the aetiology of the addiction problem [2,3].

The addiction symptoms related to ‘*unhealthy preoccupation*’, usually defined in and translated into items within psychometric scales, has been considered oversimplified with regards to its content and the structure of problematic beliefs and assumptions that govern online behaviours producing harm for the user [4]. Therefore, preoccupation is an addiction symptom which needs to be revised to consider multiple ways in which it is defined and evaluated. There are multiple cognitive factors underlying preoccupation which are not usually considered within existing measures. For instance, erroneous beliefs about probabilities in the case of addictive online gambling is one of the specific cognitive elements, which is not always included in the items assessing this addiction problem.

Nowadays, CBT interventions are applied ranging from brief and low-intensity to long-lasting and high-intensity interventions often used for drug addiction problems, and a few are being tested in behavioural addictions concerning technology use (e.g., gaming disorder). Most individuals presenting with both substance-based and non-substance-based addictions have co-occurring mental health disorders, past traumas, and/or some level of functional impairments (e.g., cognitive, social) which may predict treatment outcome and appear to differ between addiction problems [5,6]. CBT is regarded as a ‘gold standard’ of psychotherapy in the treatment of most mental health including internet use-related addiction disorders [7,8]. However, current meta-analyses in the field of substance use disorders show that CBT can only be considered a preferred option for patients with comorbid anxiety and depression [9,10].

In the case of internet addiction (IA), CBT-IA developed by Young [11] was the first psychological treatment framework for Generalized or Specified IA problems (GIA or SIA). It is based on recognising users’ feelings, thoughts, beliefs, and actions and alleviating them within a short timeframe of 12-weekly sessions and a six-month follow-up, while learning new internet usage patterns and coping skills to prevent relapse. The programme includes three phases: (i) behaviour modification (e.g., if a user is addicted to online pornography they are encouraged to learn how to refrain from using these websites while still being able to use the internet for other purposes), (ii) cognitive restructuring (e.g., gamers who use games to build self-esteem will begin to understand that they may be gaming to satisfy unmet needs in their offline life), and (iii) harm reduction therapy (e.g., examining the issues that led to the addictive behaviour to find healthy solutions).

Previous research on CBT to reduce IA has shown that what is relevant for the user with an online addiction problem is understanding and evaluating the problematic event. In other words, cognitive reconstruction can support the transformation of the user’s negative thoughts to incorporate new meanings, feelings, and behaviours associated with online activities [7]. The modification of the cognitive and behavioural sphere that supports the unhealthy online habit requires a change in cognitive style (i.e., reconstruction), development of healthier and adaptable skills (i.e., skill-building), and acquisition of a new lifestyle (reorganising daily life). In more in-depth approaches, such as the CBT-IA [7], the aim is to identify the underlying aetiology of IA by designing a plan to tackle the practical problems concerning the online habits so that IA symptoms can be improved. From a research perspective, not all the systematic reviews focussing on IA symptoms and psychology are congruent in the effects of CBT on GIA and SIA (e.g., such as time spent in gaming), as there is limited research concerning the psychological components related to internet use-related addiction problems.

To date, only a few studies have attempted to describe what the thoughts, beliefs, feelings, and associated behaviours of individuals prone to developing GIA or other specific forms of online harms are (e.g., gaming disorder, online gambling disorder, social media addiction, cybersex addiction, and online shopping disorder). Thus, the present study explored the cognitive components that according to users can lead to a potential internet use-related addiction problem using an adult European community sample.

## 2. Materials and Methods

### 2.1. Study Design and Participants

An exploratory mixed-methods design was used, specifically a ‘QUAN + qual’ design (according to Morse nomenclature [12]). The notation proposed by Morse (1991) is useful for representing mixed-methods designs because the system uses the abbreviations ‘quan’ and ‘qual’ to represent the quantitative and qualitative elements of a study. When one method has greater weight than the other, it is described using capitals letters (e.g., QUAN + qual means the quantitative component has greater weight). The symbol ‘+’ indicates a simultaneous design [13]. The expansion purpose refers to seek to analyse and explore different facets of a phenomenon to obtain a fine-grained understanding of it. In the present mixed-methods study, the design with the methodological expansion aim pretends obtain an in-depth understanding of the cognitive component underlying the quantitative measures of possible online compulsive users to know their perceptions on the addiction problem [13]. Therefore, it was based on an online survey with a number of questions including sociodemographic variables (e.g., gender, age, country, occupation, and relationship status), validated psychometric instruments (to assess GIA and SIAs problems, and attachment styles), and a set of open-ended questions regarding the beliefs, thoughts, feelings, and behaviours related to technological addictive behaviours.

The survey included a convenience sample of 854 adults (aged between 18 and 79 years old; mean (*M*) age of 26.7 years [standard deviation (*SD)* = 11.1]) comprising 175 men and 679 women from European countries mainly from Belgium (*n* = 343), France (*n* = 262), Spain (*n* = 109), Switzerland (*n* = 61), United Kingdom (*n* = 31), and Norway (*n* = 28). The participants voluntarily answered an online survey, in one of three languages (i.e., French, English or Spanish), that was advertised in the context of the Marie Curie ‘Tech Use Disorders’ project (https://cordis.europa.eu/project/id/627999) (accessed on 6 November 2021) [14] through advertisement across several European Universities based on the co-authors’ affiliations. Therefore, 73.9% of the sample comprised university students that were either single (57.8%) or in a relationship (27.2%), with an education level between secondary and tertiary education (33.8% and 53.4%, respectively).

### 2.2. Measures

#### 2.2.1. Psychometric Scales for the Quantitative Part of the Study: Self-Report Measures

The Compulsive Internet Use Scale (CIUS [15]) includes a total of 14 items rated from 0 ‘never’ to 4 ‘very often’. Scores range from 0 to 56, with higher scores referring to greater GIA severity. A couple of cut-off points have been selected: a tentative score of 21, which has been tested [16] instead of the original 28 (i.e., 14 items × 2 ‘sometimes’ = 28 [17]), and a conservative score of 42, as here our interest was on selecting those who had at least provided ‘often’ as usual response (i.e., 14 items × 3 [‘often’]) as possible diagnosis. The original CIUS showed adequate factorial, content, and concurrent validity, and satisfactory reliability (i.e., Cronbach’s alpha). The language adaptations used in the present study were previously validated [18]. In the present study, the word ‘internet’ was also changed to ‘online video gaming’, ‘online gambling’, ‘social networking sites (SNS)’, ‘cybersex’, and ‘shopping online’ to assess these other specific compulsive usages. Therefore, each participant provided answers to as many CIUS adaptations in relation to online uses that they were engaged in. Their reliabilities (i.e., Cronbach’s alphas) ranged between 0.90 for GIA and cybersex, and 0.94 for gaming.

The Relationships Questionnaire (RQ [19]) is a 4-item scale designed to assess adult attachment styles through one item per relationship style using a 7-point Likert response. The styles are secure, dismissing-avoidant, fearful, and preoccupied (i.e., the only single item was “*I want to be completely emotionally intimate with others, but I often find that others are reluctant to get as close as I would like. I am uncomfortable being without close relationships, but I sometimes worry that others don’t value me as much as I value them*”). An individual may rate a combination of styles, usually one being higher than the others, indicating a profile of the participant’s attachment feelings and behaviours. It is commonly used to identify the underlying attachment dimensions from linear combinations of the individual prototype ratings or through a composite attachment measure: positive self-model ([secure + dismissing] − [fearful plus preoccupied]), or anxiety model reversing the equation ([fearful + preoccupied] − [secure + dismissing]).

#### 2.2.2. Open-Ended Questions for the Qualitative Part of the Study: Self-Perception and Opinions

The survey asked a set of questions regarding various aspects of internet use:

- Do you sometimes feel dependent on activities that you perform on the internet? If yes, how did this dependence manifest?
- Many people have experienced a period when they excessively engaged in online activities. Has this happened to you? (Yes/No, self-perception of the addiction problem)
- What was this activity? Videogames, social networks, video streaming, etc.
- Can you describe this period?
- What were the causes?
- How did this activity gain importance over other activities of daily life?
- What (or who) were the events or people that made you realise that you engaged in your favourite online activity excessively, and how?
- What was the duration of this period of excessive use?
- Did you seek the help of a professional related to your problematic use of online activities? Yes/No. If yes, what type of activity/ies does this refer to?
- During the period in which you engaged in internet use excessively, could you mention other psychological problems you may have experienced? For example, anxiety, etc.
- If you have been able to control or stop excessive internet use, how did you cope? For example, when the main problem has been solved, did you replace the activity, etc.?
- What kinds of emotions did you feel during this period?
- What does your favourite online activity mean to you?

### 2.3. Data Analysis

In the quantitative data analysis, compulsive usages of different online activities (i.e., GIA and each SIA) were first explored using the *M*, *SD*, range (maximum score–minimum score), the tested cut-off point (21)—of ‘sometimes’ engaging in compulsive usages, and the conservative cut-off point (42)—of ‘often’ being online compulsively. Similarly, the attachment styles (i.e., secure, dismissing, fearful, and preoccupied) were described. Subsequently, Pearson’s correlation coefficient (*r*) was used to explore the relationships between the CIUS adaptations and attachment styles (RQ). An exhaustive chi-squared automatic interaction detection (CHAID) was applied to detect mutually exclusive subgroups in the sample that differed regarding GIA (the criterion variable used was whether or not individuals experienced compulsive internet use, demarcated with a cut-off point of 42), and predictors were the categorical variable of self-perceived addiction problem (yes/no), and the three less healthy attachment styles (i.e., dismissing, fearful, and preoccupied), measured continuously. This technique selects the best predictors of the outcome, dividing the sample into subgroups based on the initial variable, merging non-significant categories and considering the interaction between the criterion and predictor variables. Student’s *t*-tests were used to determine whether self-perception of dependence on using the internet at least once in life was influenced by general and specific internet usages. IBM SPSS 24 software was used. (IBM SPSS Statistics for Windows, Version 24.0; IBM Corp: Armonk, NY, USA, 2016.)

In the qualitative analysis of the open-ended questions, a descriptive content analysis technique was used to process these data according to Krippendorff guidelines [20] at the semantic level due to the shortened responses gathered from those users classed according to the CHAID results. In other words, cognitions that were related to compulsive internet use in those users who had a higher probability of developing GIA were observed.

### 2.4. Ethics

The Ethical Committee of the Psychological Sciences Research Institute, Catholic University of Louvain (UCLouvain) approved the study protocol. Participants provided informed consent and voluntarily participated following the assurance of confidentiality and anonymity (see Appendix A).

## 3. Results

The sample’s characteristics regarding potential compulsive internet use, comorbid psychopathology, attachment styles, and models are provided below.

### 3.1. Quantitative Findings

#### 3.1.1. Compulsive Internet Use

The following scores were obtained from the sample across the six CIUS versions (see Table 1).

Almost all the sample voluntarily filled in the original CIUS, and the scale adapted to the use of SNS, which appeared to be the common online behaviours. Potential GIA had a prevalence of a third of the sampled individuals, who perceived they engaged in compulsive use ‘sometimes’, and only 2% stated that they engaged in compulsive internet use ‘often’. Similarly, regarding SNS use, almost a third reported engaging in compulsive use ‘sometimes’, and almost 3% ‘often’ engaged in compulsive use. Regarding the other specific online activities, ordered from higher to lower number of responses, with regards to online shopping, 5% of the sample reported they ‘sometimes’ engaged in compulsive buying behaviour, and only 1% considered this to be compulsive ‘often’. The use of videogames was the fourth activity with almost 60% of the sample self-identifying as gamers, with approximately 17.4% gaming compulsively ‘sometimes’ and 1.2% problematically. Cybersex was engaged by almost half of the sample, with about 4% reporting to ‘sometimes’ engage in compulsive cybersex, while no participant perceived it to be an addiction problem. Lastly, gambling was engaged by less than half of the sample (42%), of whom 5% indicated they ‘sometimes’ gambled compulsively, and 1% ‘often’.

#### 3.1.2. Attachment Styles

The attachment styles present in the sample are described in Table 2.

In this sample, almost all participants responded to the RQ scale. All four styles were almost equally present with the most positive ones slightly more prevalent according to the self-model: secure, dismissing, and fearful [21].

#### 3.1.3. Relationships between Compulsive Internet Usages and Attachment Styles

In Table 3, association between almost all CIUS scales are presented, ranging from moderate positive correlations (i.e., GIA and SIA for SNS, SIA for cybersex and gambling) to low level associations (e.g., SIA for gaming and gambling), and a case of no association (i.e., GIA and SIA gambling). Regarding attachment styles, the secure type did not correlate with any compulsive problems, nor did the dismissing style (only with a small association with GIA, *r* = 0.09, *p* < 0.05). However, the preoccupied attachment style was related to almost all problematic internet usages, except gaming and shopping compulsively, and fearful attachment style related to GIA, SIA with SNS, and shopping with small correlations.

#### 3.1.4. Chi-Squared Automatic Interaction Detection

**Figure 1:**
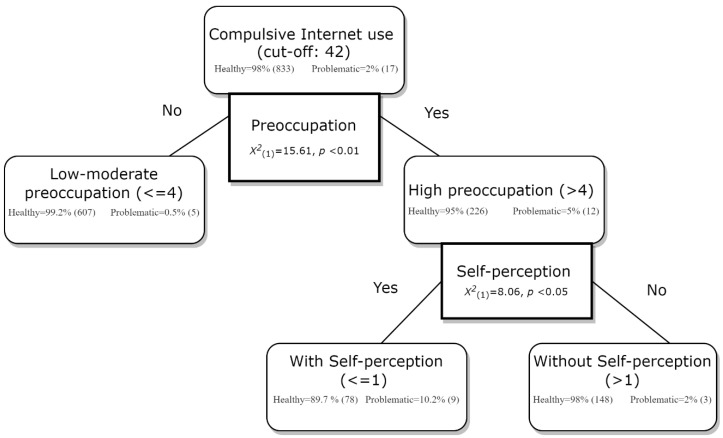
Discriminating factors for compulsive internet use in adults (subgroups of healthy and problematic users—with percentage and frequency).

Results of stepwise CHAID for the discriminative factors for GIA are shown in Figure 1. This decision tree explored data creating profiles through automatic detection of interactions between the relevant variables using chi-square. In the present sample, this technique showed that high preoccupation style was the first and most significant predictor distinguishing between adults with and without compulsive internet use (using the cut-off of 42). Subsequently, among those with a preoccupied attachment style, presenting with self-perception of the addiction problem was a second discriminating predictor for compulsive internet use.

In summary, the highest probability of developing compulsive internet use (i.e., 10.2%) was found in European adult users who had a preoccupied attachment style and self-perception of the addiction problem, while the lowest probability (0.5%) was found among those with a moderate or low preoccupied attachment style.

#### 3.1.5. Association between Self-Perception of Dependence and Compulsive Usages

Student *t*-tests showed that users who recognised having felt dependence on using the internet at least once in their lifetime (i.e., self-perception of the problem) were usually classed as presenting with potential: GIA (original CIUS: *t*_(488)_ = 7.52, *p* < 0.001), and/or a SIAs (CIUS-gaming: original CIUS: *t*_(316)_ = 6.32, *p* < 0.001; CIUS-SNS: *t*_(489)_ = 2.9, *p* < 0.01; CIUS-Cybersex: *t*_(255)_ = 2.58, *p* < 0.05).

The self-perception variable was used for the qualitative analysis together with a variable classifying participants with potential compulsive internet use (i.e., cut-off point of 42), and preoccupied attachment style (scoring > 4 out of 7). For the qualitative analysis, cases were filtered based on CHAID results to ascertain the perception of users who appear to be at risk of addiction and are aware of the cognitive components, as theoretically these individuals can provide more insightful information.

#### 3.1.6. Content Analysis of Potentially Internet-Dependent User Accounts

**Figure 2:**
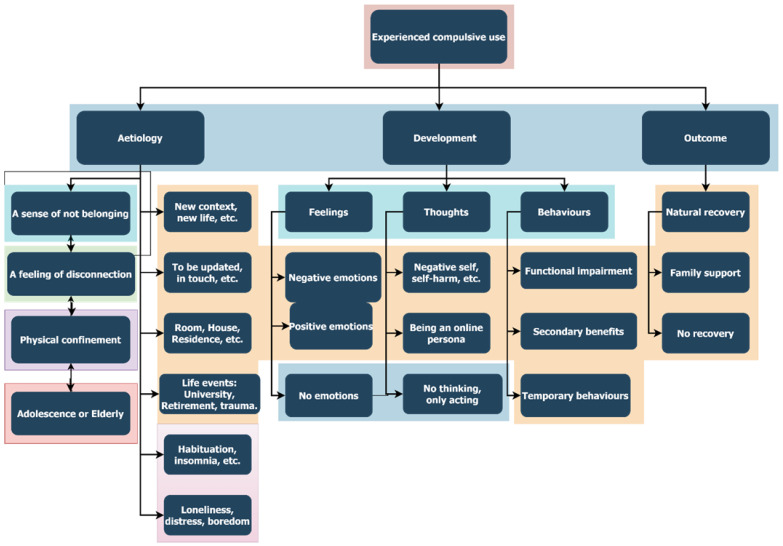
Themes and subthemes of the qualitative analysis.

The subsample of these extreme compulsive users comprised nine women (age range was 18 to 73 years [*M* = 27.44, *SD* = 17.39]), who presented with SIA. Three had engaged in compulsive gaming, three in compulsive social networking, and one in compulsive shopping. There were five undergraduates, three employees, and one retired person. Four had a partner while five were single (the oldest participant was divorced), and three had completed university studies, while six had not reached this education level yet. The users who classed themselves as using the internet compulsively often admitted their activities were (ordered by frequency): social network use (i.e., Facebook and Twitter), videogame playing (i.e., MMORPG), video streaming, and other chat applications. The main results are presented in Figure 2.

The main meaning and illustrative quotes of each subtheme are presented in the following subsections, in which the detail of each subtheme will be included in a list of bullet points with a few illustrative quotes. Each participant has as code as an identifier according to their ID number (ranging from 1 to 9) and age (in numbers). For instance, P126 represents Participant number 1 who is 26 years old.

##### Theme 1: Aetiology of Compulsive Internet Use

The origin and the causes of this maladaptive internet use pattern according to the problematic users were based on feelings rather than thoughts or behaviours.

- A sense of not belonging in their daily environment—e.g., first years at university, retirement, or trauma.
  ○ *“Feeling like I had nothing in common with the people I met in class I decided to start playing online and meet a lot of people with the same passions as me except that the time zone prevented me from playing, talk to them as often as I would like”* (P619)
  ○ *“I knew my mother had breast cancer. I was very alone, and I did not find any recomfort from my friends, who did not understand my state of sadness. I did not have the courage to tell them about it, and I slowly closed myself and looked for being comforted online through a forum”* (P519)
- A feeling of disconnection: A feeling of disconnection was compensated for by online connection through updates, notifications—i.e., continuously looking for connection with others in games, following others’ lives on SNSs, or through watching online series (e.g., binge watching). Users stated that during the period they felt online use was addictive they were emotionally distressed (e.g., angry, stressed, depressed) or lonely. Using social networks on smartphones facilitated their online habits. These two quotes illustrate this subtheme:
  ○ *“The addiction may not be due to a chemical substance (as may be the case with heroin) but rather to psychosocial components (keeping abreast of the lives of others, showing that one has an active life, having the impression of missing out on your social life if you are not on social networks)”* (P724)
  ○ *“People can gain satisfaction from ‘likes’ on photos they post, something which they may not get in reality, i.e., face-to-face compliments”* (P920)
- Physical confinement: e.g., the room, in a period with restrictions (e.g., to prepare for exams), with no responsibilities (e.g., holidays), or neglecting responsibilities.
  The following quotes illustrate the impact of physical confinement on problematic internet use:
  ○ *“I feel dependent as I do not find a rewarding outdoor activity. This manifests itself in constant research”* (P273)
  ○ *“Instead of leaving the house and doing activities outdoors, I would just stay in and remain on my computer most of the day”* (P920)
  ○ *“A getaway from life, a connection outside but at home”* (P821)
- Adolescence and the elderly: A developmental stage in the life span associated with problematic internet use: usually adolescence or early adulthood, but the elderly have emerged as denoting a possible developmental stage (i.e., retirement).

##### Theme 2: Development of Compulsive Internet Use

Regarding the development of compulsive internet use, users stated there was an acquired habit of balancing feelings, thoughts, and behaviours when using the internet:

- Feelings: the negative emotions (i.e., loneliness, lack of motivation, tiredness, anger, boredom, narcissism, stress, anxiety, depression, and suicidal ideation) disappear when online, and positive emotions take their place (e.g., having fun, being happy, escaping from reality, being enthusiastic, hopeful), but when offline, they return (e.g., “*It was going from one extreme to another*”) in a cyclical pattern (i.e., escapism). Neutral emotions or the absence of them have led to a compulsive act. Illustrative quotes are the following:
  ○ *“I feel a lack in me until I satisfy it (online) and it makes me irritable, nervous, stressed and a little lost and wanton”* (P318)
  ○ *“This excessive use definitely got worse the more followers I gained”* (P920)
- Thoughts: During the problematic internet use period, participants’ thoughts related to negative self-perception: e.g., less concentration; insomnia. Participants decreased the frequency and quality of other responsibilities, which became secondary in the users’ life, and the online activity (e.g., SNS use) became the primary activity in users’ lives (i.e., increased salience). Participants felt a desire for being admired as an online persona whilst not feeling important offline. Indeed, they expressed they did not engage in much thinking, but instead engaged in using the internet compulsively. The link between positive feelings and negative thoughts regarding the insight into the problematic online habit is illustrated by these quotes:
  ○ *“Sometimes we realise it, but it’s difficult to do something about it, because these (online activities) continue to convey positive feelings to us despite everything they generate alongside”* (P519)
  ○ *“These activities allow you to get away from it all and think about something else. If I am not on the internet, I will quickly get bored and find nothing to do and therefore, return to the internet”* (P821)
- Behaviours: Participants recognised functional impairment affecting studies, work, social and family life, even hobbies (e.g., sports). As engaging in online activities at home reduced sleep time and concentration on other tasks, it slowly created online habits to the detriment of offline habits. The online actions became central behaviours in the participants’ daily lives. Participants obtain secondary benefits, such as coping with negative emotions and thoughts. Some of the relevant quotes were:
  ○ *“I put aside my work to check my emails, answer my emails, go to social networks”* (P126)
  ○ *“I stayed up late at night to tune in to the American time zone and so in class I felt a lot of fatigue and difficulty following the lessons”* (P619)
  ○ *“Day after day, it has taken a central place in my daily life until it punctuates my days. I only go out to go to college, I often tell myself with hindsight that the best phrase that defines my day these days is “I vegetate”* (P318)

##### Theme 3: Outcome of Compulsive Internet Use

The usual duration of the compulsive internet use period was between a year and three years, and none of the participants looked for any professional support (e.g., psychologist or psychiatrist). However, all confirmed having suffered psychological problems such as depression, anxiety, social phobia, and other addictions.

The outcome was:

- No recovery: Half of the sample was still experiencing problems at the time of the study.
  ○ *“In progress. Started with the regular use of my smartphone as soon as I have nothing else to do, to turn into smartphone use even when I have other things to do”* (P724)
  ○ *“I realise this already by the time spent on the computer or other (device), but also when one is deprived of the internet, one seeks to recover it. If the computer is down, we will go to the smartphone all day or the TV, etc.”* (P821)
- Recovery with family (and friends’) support: A quarter of the sample received support from family members. Friends usually alerted them and flagged some of their problematic behaviour (e.g., participants received nickname such as ‘R2-D2’ because they were perceived to be hyper-connected by their friends), and family (e.g., sister, sons/daughters, parents, partner), and one user stated her parents could not help her.
  ○ *“My partner has often pointed it out to me”* (P126)
  ○ *“My children, a friend”* (P273)
  ○ *“No one since I live alone, if I still lived with my parents I would never have arrived there, my parents would have slowed me down! It is precisely the fact of living alone that allowed me to fall into this infernal circle, since I am going from an activity that I enjoy to a real problem. When I was a high school student, my mother told me to control my time on the internet, I had freedom, but she kept me from going overboard”* (P318)
- Natural recovery: The other quarter of participants had naturally adjusted their compulsive internet use. Their problematic use was not only characterised by the amount of time they spent online, but also by craving they experienced when not online (indicating possible withdrawal). Functional impairment also made them realise their problem: they failed courses, lost a job, a partner, or had negative health outcomes (e.g., insomnia, depression). The mechanisms they used to help themselves were to manage online time and engage in outdoor activities (e.g., going out with friends to the cinema, to the restaurant, engaging in sports or excursions) or looking for new activities to engage with at home by themselves (e.g., cooking, playing music or reading books). The main quotes illustrating this are presented below:
  ○ *“When you cannot use the internet, for example because the computer is broken, you go to connect through the smartphone all day”* (P724)
  ○ *“Reading provides me the same sensations as my addiction, what I look for escaping, and to be in a world that is not my reality; I hope reading will support me to reduce and replace my online activity”* (P318)

The differently weighted concurrent design (QUAN + qual) provided greater weight to the quantitative study. This is because the sample size and analyses undertaken were needed to scientifically filter the targeted cases employed in the qualitative analysis. This resulted in a small and evidence-based sample from a theoretical perspective. The simultaneous collection of information at the same time within the survey was analysed following an analytical strategy. Firstly, statistical techniques provided the description, estimation of the prevalence of the constructs measured, the association between these constructs, and the extraction of the risk predictors of compulsive internet use by groups to qualitatively study the most affected group (i.e., those with a preoccupation style and self-perception of the problem). In other words, the methodological aim was managed through two stages. First, the scales focused on fixed characteristics of the overall sample, and second, the open questions addressed dynamic aspects related to the generation, development and management of the online behavioural problem of those who were potentially the unhealthiest compulsive users according to the quantitative analysis. This provided insight into the cognitive components of those most potentially affected.

## 4. Discussion

The present study explored the cognitive (thoughts and emotions) components that can lead to internet use-related addiction problems through potential dependent participants. The aim of the present study was to develop a deeper understanding of individuals’ experiences to support tailoring future CBT interventions which can target underlying cognitive components to develop prevention approaches.

The internet-use related addiction problems which appear at the root of these behavioural problems, according to the study’s findings, are usually GIA, the addictive use of social networks and gaming. The estimated prevalence of these problems in the present European sample of adult users was approximately 2%, which seems consistent with previous European reviews (in which the reported prevalence was 2% [22,23]), except an empirical study in which the minimum prevalence was 15%, which can be explained by the researchers using a different scale with a lower cut-off score [24]. However, the emergence of social networking has not always been found to be problematic in the literature. A review of internet use-related problems in Europe indicated that there is sufficient scientific evidence only for GIA, problematic gaming and gambling as potential IA using the applied reviewed criteria, and these problems require developing prevention strategies and policy options [23]. This latter aspect of prevention strategies is an educational component which could be embedded into CBT interventions, especially those which include family members in a systemic approach to learn about this health condition and treatment options together (e.g., partner, parents, adult children), as the present research has indicated the importance of social support for recovery.

CBT with ACT can include significant others e.g., using acceptance and mindfulness strategies with commitment and behaviour change strategies to increase psychological flexibility—i.e., providing “functional contextual” meanings in the adult relationships or parenting. Functional contextual meanings refer to the conception that psychological events are ongoing from a contextual point of view [25]. According to this premise, the addictive behaviour can have a different function for each individual, which is the reason why behaviour change can be accomplished through modifying the environmental factors (i.e., “contextual meaning”), in which family can have a role while the individual is developing new processes to not let feelings and thoughts exacerbate the addiction cycle. Accordingly, an ACT clinician might explore how a specific stream of thoughts functions for a patient to manage this process in analytical terms (e.g., What does the person gain or lose from engaging online compulsively? What function does the online behaviour have for the person? Why has internet use led to the emergence of problems?).

In general, online addiction problems yielded small to high positive bivariate correlations (between *r* = 0.15 and *r* = 0.57), which is higher than those indicated in previous studies [3,25], except for GIA and online gambling addiction, as well as gaming and social networking addictions, for which the present study did not find an association. A recent study focused on gaming found internet-based problematic behaviours were separate entities [26]. Therefore, in CBT applied to GIA, comorbidity with other psychopathology and other online problems should be assessed and potentially prioritised depending on the respective client’s individual problem constellations [8], as relieving emotional distress can indirectly reduce problematic internet use [6].

Attachment styles are relevant dispositional factors in determining online addiction behaviours. In the present study, the unhealthiest style, preoccupied [19], was slightly less prevalent than the other attachment styles. According to the literature, the preoccupied style seems to be related to increased risk of GIA [27,28], social networking [29], gambling [30], and cybersex [31] addictions. The reasons for preoccupation with internet use are diverse but related to the absence of other people or activities which can help balance online and offline activities. Compulsive SNS use seems to be a way of replacing and compensating for missing affection from family, partner, or peers [32] (i.e., gratification model). Individuals affected by the compulsive use of cybersex, as stated in the literature [31], seem to maintain a sense of unworthiness or feeling unlovable, and tend to strive for the acceptance of others as a means for attaining self-acceptance. In other words, the healthiest attachment styles (i.e., secure or dismissing) can be protective factors and their derivatives may be included as strategic components to enhance in CBT interventions, such as self-esteem, autonomy, social support, prosocial coping skills, ability to share emotions with others, impulse and feelings management, trust, intimacy, affection, resilience, and long-term friendships.

Regarding the findings from the CHAID analysis, this explorative technique scarcely used in the field of addictive behaviours, has identified other predictors apart from depression, which was identified in a previous GIA study [33]. In the present study, self-perception as insight into the problem and preoccupied attachment style were the main predictors to differentiate those with higher GIA versus lower GIA. Therefore, preoccupation as an addiction symptom requires further research [4] linked to the attachment styles. The present study allowed to gain in-depth knowledge about the underlying cognitions of those classed as potential users with an addictive behaviour engaged in through the internet and associated attachment styles.

Concerning the qualitative findings, present beliefs associated with negative thoughts (negative self-appraisal) of those categorised as potentially addicted users helped shed light on the aetiology of these problems. Therefore, the causes of their problematic internet use as perceived by users are the main elements to address in harm reduction therapy. This is how CBT interventions can be individualised (e.g., assessing the extent to which one of the causes of problematic internet use was based on perceived isolation due to leaving home to start university studies, and understanding what caused the perceived lack which was filled by means of engaging in online activities, which facilitated escaping from the discomfort experienced offline). The qualitative findings suggest that for some users, the addiction problem is an adaptation problem when a life event occurs, and the person has limited alternative coping strategies (or persons to support them).

Problematic users have experienced disconnection from others and their life has changed in a way explained by internal and external factors (e.g., non-adaptation, leaving home to start a university degree, or leaving work life for retirement, experiencing a painful period due to a trauma—e.g., severe illness of a loved one, loss of a loved one, which seems to produce an enclosure on oneself). Although adolescence and early adulthood are usually targeted as the main developmental stages in which problematic internet use occurs, an older developmental stage has also been identified [34], which is usually associated with loneliness [35], similarly to the experiences of adolescents and early adults. Therefore, CBTs should be open and tailored to clients of any age, as the problem can be linked to life events which make people susceptible to experiencing isolation. Consequently, more research on older users is necessary.

In CBT, unhealthy learned pathways need to be unlearned [2,3]. This form of therapy can help by understanding the users’ initial motivation to use the internet in the first place. Unhealthy usage patterns can then be changed progressively by managing associated thoughts, beliefs, and feelings. Healthy and more mindful usage of the internet can then be re-established through behaviour modification strategies [8]. However, to change the meaning and actions related to the management of their feelings and creating a vicious cycle (e.g., using the internet to change mood temporarily, escape from the stresses of reality) of using the internet should be a priority, while managing complementary intervention actions (e.g., improving self-esteem, learning how to cope with boredom or sadness in healthier ways). Furthermore, CBT should integrate the psychosocial ingredients to support the patient on learning alternative prosocial skills and find individuals and activities outside the online environment.

Alternatively, ACT suggests “cognitive diffusion” (i.e., the process through which a person comes to understand that their thoughts are verbal events rather than actual events) can substitute the unlearnt pathway [25] if is too difficult to unlearn it. In other words, instead of trying to change the contents of users’ thoughts, it can modify the function of user’s thoughts through changing how the user interacts with them (e.g., to learn how to cope with uncomfortable feelings and thoughts attached to social network use by diminishing their status in the user’s perception to let other healthier alternatives emerge).

More importantly, to disconnect the established habit (i.e., the compulsion) requires integrating the cognitive component while making life changes, such as engaging in outdoor activities, as indicated by the potentially dependent users studied who seem to naturally have recovered by themselves or through family support. The underlying cognitions associated with online addiction need to be established, e.g., What is the motivation for and the need to be constantly connected to the SNS or to games? Which internal aspects (e.g., trauma, insomnia) play a role in establishing the habit? Which external factors play a role in the unhealthy habit (e.g., confinement, retirement)? Which factors are relevant in the development of the habit according to the user? Which alternatives have not worked to reduce compulsive internet use and why?

It is noteworthy that most participants in the present study were adult women, which is similar to the only European empirical conducted on adults [24]. Therefore, on this continent (and probably for Western cultures), health providers and clinical researchers should target addiction in women as the emergent literature is showing, for instance, female gamers have specific addiction patterns [36,37].

If the path to recovering wellbeing is unlearning the addictive behaviour pathway, mixed emotions regarding the addictive online activity can be key factors associated with users’ thoughts. Similarly, actions that cause harm (e.g., functional impairment) and flag health problems should be taken into account to meet CBT goals (i.e., mindful technology use). In a few cases, GIA can be a mechanism to take advantage of (secondary benefit), as the primary benefit can simply be to escape from reality, or compensation for other life problems (e.g., school stress, personal emotional distress). It may be easier for problematic users to talk about their feelings rather than their beliefs or thoughts regarding their addictive habits. However, the most worrying case for CBT interventions refers to those users who do not have problem recognising their patterns of problematic usage, as indicated in the present sample. None of these users has looked for health support, although they were aware of other comorbid mental health problems. It is alarming that half of the participants affirmed maintaining excessive internet use (i.e., they did not recover), despite having stated that they were aware of their addiction problems.

In the field of IA, studying their development and recovery is still in its infancy, as recently indicated by Zajac and colleagues [38]. There is almost no research on the natural progression of these conditions, making it difficult to determine when and how much treatment is necessary to improve natural recovery rates. More understanding of compulsive internet use from a bottom-up perspective is needed to be able to establish efficacious interventions. Only a few studies have started to qualitatively investigate the phenomenology from potential problematic users [3,39] and clinicians [40]. Previous studies have shown that CBT is the most effective and efficacious approach for treating internet use-related problems [6,8,9,11] and have provided a detailed CBT-based therapy programme to treating internet use-related disorders [8,11]. However, in comparison with the substance use disorders field, there is not yet enough evidence to consider CBT as a ‘gold standard’ in the behavioural addiction field, as the clinical literature in both addictions (substance and behavioural addictions) is growing offering new psychological and pharmacological interventions that are showing efficacy in treating addiction problems [6,41,42]. Nevertheless, the present research has showed that it is important to target the emotional components and cognitive restructuring when associated with online addiction problems (e.g., individuals with problematic online shopping can benefit from group counselling [43,44]).

Despite the many strengths, one of the main limitations of the present study is that the findings were derived from a voluntary, non-random sample of European users recruited from academic settings using self-reported methodology, and predominantly female which may explain the low prevalence of gambling and cybersex addiction problems (e.g., due to social desirability). However, the mixed-methods design adopted, alongside validated psychometric tests, and assessment of online addiction, and attachment styles has provided a fruitful strategy to identify a subset of the sample in order to analyse problematic adult users’ perceptions qualitatively. These users were potentially dependent, were aware of their problems, and had a preoccupied attachment style. In future studies, more contemporary attachment style scales could be used that include validated items to identify the preoccupation style, as this study has identified a particular attachment style, preoccupation, associated with problematic internet use. Some of the literature discussed in this section has provided valuable knowledge on addiction problems, which is uncommon in the field. For future in-depth research analyses regarding the role of the preoccupation attachment style—which has become a focus in the IA field [27,28,29,30,31]—it is recommended that researchers try to use the longest version developed by Griffin and Bartholomew, the Relationship Scales Questionnaire [45].

It should also be noted that the present study provides limited conclusions regarding men with possible GIA as there were no male data in the qualitative samples extracted through the CHAID analysis. This may have been due to the imbalanced gender convenience sample (which had significantly more women) and/or due to the predictors extracted by the CHAID analysis (i.e., high preoccupation, self-perception), or even the average age of the adult sample. The literature in the field clearly shows that males have higher prevalence of SIAs compared to females, such as online gambling, online gaming, and cybersex. Finally, no causal mechanisms could be established due to the study design employed because CHAID analysis is an explorative statistical technique. In other words, with some caution, the present findings can be used to support the development of future CBT strategies and to add to existing CBT protocols aimed at treating IA [8].

The next research steps to develop a research agenda concerning IA that arise from the present findings can be summarised in ten actions:

(i) There is a need to further study not only GIA but specific online addictions that are present among those potentially categorised as problematic users;
(ii) To perform naturalistic prospective follow-up studies examining the course of IA;
(iii) To develop evidence-based prevention strategies including educational components for a variety of stakeholders (i.e., peers, family, and carers);
(iv) Future psychological assessment needs to include the spectrum of online addiction problems to strengthen the body of knowledge and to help develop more tailored interventions; including an understanding on preoccupation attachment style as;
(v) Given that some users said they had difficulties in adapting to new life events (e.g., adolescence, retirement) and/or felt disconnected, pathological use of the internet use appeared to be a coping response and requires further study to prevent the development of a full-blown addiction;
(vi) There is a need to develop clinical research with patients to continue analysing the cognitive component in CBT treatments, in which the prosocial and contextual components can possibly be key factors in the recovery;
(vii) Concerning different recovery pathways (i.e., natural recovery, recovery through social support, recovery through an out-patient clinical treatment or an in-patient clinical treatment), particularly as none of the possible compulsive users studied approached any health provider for their self-perceived problematic internet use, is necessary to know what can be the main obstacles for treatment;
(viii) Women should be targeted for research into GIA and some of the SIAs, as the emergent literature in the field indicates that problematic internet use is not just a problem among males [3,37,46];
(ix) To disentangle the underlying mechanisms in these addiction problems, including users without self-perception of the problem and those with other attachment styles (e.g., fearful style),
(x) To examine the natural progression of these unhealthy habits as well as natural recovery because they are still not well known or understood.

## 5. Conclusions

A targeted CBT intervention can be designed based on the individual’s emotional, developmental and contextual factors combined with emerging behavioural and cognitive characteristics [6,10,47]. In this European study, almost all online activities were common among adults, most of whom engaged in them in a healthy way. However, a low prevalence of online addiction problems was found in the present research. Associations between online problems and attachment styles emerged and the predictors for compulsive internet use were identified (i.e., awareness of the problem and having a preoccupied attachment style). Users with these characteristics reported how perceived isolation during life events, together with accessibility and emotional management, appeared to be at the root of establishing a potentially pathological habit, which in half of the cases disappeared naturally after a certain period or with the support of significant others. The present study also indicated that there was not sufficient knowledge available with regards to clinical support, which means more awareness-raising campaigns and prevention initiatives are needed in the field.

## Figures and Tables

**Table 1:** Descriptive statistics of the six Compulsive Internet Use Scale (CIUS) versions.

CIUS	*n (%)*	*M* (*SD*)	Range (*max–min*)	*f*_Prev1_ (%) ^1^	*f*_Prev2_ (%) ^1^
Original CIUS	851 (99.7)	17 (10.7)	51 (51–0)	289 (34)	17 (2)
CIUS-Gaming	506 (59.3)	8.8 (11)	51 (51–0)	88 (17.4)	6 (1.2)
CIUS-Gambling	354 (41.5)	0.7 (.3)	42 (42–0)	1 (0.3)	1 (0.3)
CIUS-SNS ^1^	851 (99.7)	14.8 (11.5)	56 (56–0)	218 (27.9)	21 (2.7)
CIUS-Cybersex	419 (49.1)	3.1 (6.7)	48 (48–0)	17 (4.1)	0 (0)
CIUS-Shopping	692 (81)	5.1 (7.1)	48 (48–0)	34 (4.9)	1 (0.1)

^1^ Note: SNS = Social networking sites; Prev1 used cut-off point of 21, Prev2 used cut-off point of 42; Frequency (*n*) and percentage of responses (%), mean (*M*) and standard deviation (*SD*), range of scores (maximum score–minimum score), frequency (*f*_i_) and percentage (%) of the two estimated prevalence (Prev).

**Table 2:** Descriptive statistics of the Relationships Questionnaire (RQ) Test.

RQ ^1^	*n (%)*	*M* (*SD*)	Range (*max–min*)
Secure	851 (99.7)	4 (1.7)	6 (7–1)
Dismissing	851 (99.7)	3.7 (1.9)	6 (7–1)
Preoccupied	847 (99.5)	3.3 (1.9)	6 (7–1)
Fearful	851 (99.7)	3.7 (2)	6 (7–1)

^1^ Note: Frequency (*n*) and percentage of responses (%), mean (*M*) and standard deviation (*SD*), range of scores (maximum score–minimum score).

**Table 3:** Relationship between compulsive internet use scales (CIUS) and attachment styles measured by the Relationships Questionnaire (RQ) Test.

		Correlations					
		1	2	3	4	5	6
Variables	*M* (*SD*)	*r*	*r*	*r*	*r*	*r*	*r*
1. CIUS	17 (10.66)						
2. CIUS-Gaming	8.82 (10.97)	0.33 ***					
3. CIUS-Gambling	0.72 (10.97)	-	0.20 ***				
4. CIUS-SNS	14.77 (11.53)	0.57 ***	-	0.22 ***			
5. CIUS-Cybersex	3.14 (6.72)	0.23 ***	0.39 **	0.53 ***	0.24 ***		
6. CIUS-Shopping	5.05 (7.07)	0.30 ***	0.15 *	0.34 ***	0.36 ***	0.22 ***	
Secure	3.96 (1.72)	-	-	-	-	-	-
Dismissing	3.65 (1.88)	0.09 *	-	-	-	-	-
Preoccupied	3.25 (1.91)	0.20 ***	-	0.16 **	0.21 ***	0.12 *	-
Fearful	3.65 (1.99)	0.15 ***	-	-	0.12 **	-	0.10 **

Note: *M* = mean, *SD* = standard deviation, *r* = correlation, * *p*  <  0.05, ** *p*  <  0.01, *** *p*  <  0.001.

## Appendix A

Tech Use Disorders Survey

(First web page)

General Objective of the Project:

We would like you to participate in a research project funded by the European Commission, which aims to evaluate the use of Information and Communication Technologies (ICT) of European adults. To participate in this study, you must be at least 18 years old, and fluent in the English language.

Tasks Requested:

Participation involves answering an online survey in the form of questionnaires that assess your ICT usage patterns. Some questions ask about the excessive use of ICTs, and a series of tests to assess psychological dimensions.

Anonymity AND Confidentiality:

Your answers will be treated anonymously. No data likely to be able to identify will be recorded and no information will be collected without your knowledge. The results of this study will be only used for research purposes. This study is consistent with the ethical standards for research involving human participants, under the Code of Ethics for research within the Faculty of Psychology and Educational Sciences at Catholic University of Louvain (UCL). It has received approval from the Ethics Committee of the Institute for Research in Psychological Sciences at UCL.

Voluntary Participation:

Your participation is voluntary. You may stop answering questions at any time and close the browser window, without any consequences. Your agreement implies that you accept that the information collected will be used anonymously for research purposes.

Thank you for your time.

Based on previous information about the pilot study, I agree to participate in the research project:

I authorize the use of the information I provide for scientific purposes and for the publication of research results, and I understand that this information will remain anonymous, that privacy will be assured and that no information I provide can be traced back to my identity.

I confirm that I choose to participate in this research.

I was informed that I can interrupt my participation at any time, without justification, and request the destruction of information that I have provided.
